# CmRCD1 represses flowering by directly interacting with CmBBX8 in summer chrysanthemum

**DOI:** 10.1038/s41438-021-00516-z

**Published:** 2021-04-01

**Authors:** Lijun Wang, Hua Cheng, Qi Wang, Chaona Si, Yiman Yang, Yao Yu, Lijie Zhou, Lian Ding, Aiping Song, Dongqing Xu, Sumei Chen, Weimin Fang, Fadi Chen, Jiafu Jiang

**Affiliations:** 1 State Key Laboratory of Crop Genetics and Germplasm Enhancement, Key Laboratory of Landscaping, Ministry of Agriculture and Rural Affairs, Key Laboratory of Biology of Ornamental Plants in East China, National Forestry and Grassland Administration, College of Horticulture, Nanjing Agricultural University, Nanjing, 210095 China; 2 State Key Laboratory of Crop Genetics and Germplasm Enhancement, College of Agriculture, Nanjing Agricultural University, Nanjing, 210095 China; 3 Present Address: College of Landscape and Ecological Engineering, Hebei University of Engineering, Handan, 056038 China

**Keywords:** Plant sciences, Plant breeding

## Abstract

The CmBBX8-CmFTL1 regulatory module is a key determinant in the transition from vegetative growth to reproductive development in summer-flowering chrysanthemum. However, the detailed regulatory mechanism of CmBBX8-mediated flowering remains elusive. In this study, we revealed that RADICAL-INDUCED CELL DEATH 1 (CmRCD1) physically associated with CmBBX8 through bimolecular fluorescence complementation (BiFC), pulldown and Coimmunoprecipitation (CoIP) assays. Furthermore, the RCD1-SRO1-TAF4 (RST) domain of CmRCD1 and the B-box of CmBBX8 mediated their interaction. In addition, Luciferase (LUC) assays and electrophoretic mobility shift assay (EMSAs) showed that CmRCD1 repressed the transcriptional activity of CmBBX8 and interfered with its binding to the *CmFTL1* promoter, thereby leading to delayed flowering in the summer chrysanthemum ‘Yuuka’. These results provide insight into the molecular framework of CmRCD1-CmBBX8-mediated flowering in chrysanthemum.

## Introduction

Flowering is a major stage of the transition from vegetative to reproductive growth during the plant life cycle. Plants have evolved a precise regulatory network to initiate flowering in response to various external and internal cues. Extensive studies have documented that BBX proteins work in concert with other key components of flowering to mediate the initiation of flowering in various plant species. *Arabidopsis* BBX10 and BBX19 directly interact with CONSTANS (CO) to repress the transcriptional activation of *FLOWERING LOCUS T* (*FT*), which in turn leads to a reduction in *FT* and FT-controlled gene expression and then brings about a subsequent delay in flowering^1,2^. AtBBX4 directly associates with the *FT* promoter in the presence of AtBBX32 to repress its transcription^3^. The CO-FKBP12 interaction contributes to the modulation of photoperiodic flowering, resulting in a decrease in *FT* in the early morning^4^. In addition, both BBX30 and BBX31 recruit CO into a TOPLESS trimeric complex to inhibit *FT* expression and flowering^5^. In chrysanthemum, multiple BBX proteins mediate flowering, either negatively or positively, through distinct regulatory mechanisms. CmBBX8, a member of the BBX family containing a CCT domain, directly associates with the promoter regions of *FTL1* to activate its transcription and accelerate flowering^6^. CmBBX24 and CmBBX13 negatively control flowering by repressing the expression of flowering-promoting factors^7,8^.

RCD1 is a member of the poly-ADP ribose polymerase (PARP) family^9,10^. Mutants with loss of RCD1 function are hypersensitive to reactive oxygen species (ROS) and exhibit early flowering^11–14^. The WWE domain within RCD1 functions as a specific protein–protein interaction motif^15^, and the PARP catalytic domain within RCD1 contains multiple conserved residues that are required for the formation of donor sites (Gly-347, Leu-348, Ser-375) or acceptor sites (Tyr-378). These distinct functional residues contribute to diverse cellular processes, including chromatin remodeling, genomic imprinting, and transcriptional regulation^14,16–18^. The RST domain present in RCD1 is involved in the assembly of the multimeric general transcription factor IID complex (TFIID)^13,19^. Although it has been demonstrated that RCD1 associates with the BBX protein from *Arabidopsis* in yeast, the biological consequence to flowering of this association remains elusive^13^.

In this study, we report that CmRCD1 interacts with CmBBX8 to repress the expression of *CmFTL1*, thereby resulting in inhibition of flowering in cv. ‘Yuuka’. Collectively, our results reveal a regulatory module consisting of CmRCD1, CmBBX8 and CmFTL1 in the regulation of flowering.

## Results

### Isolation and expression pattern of *CmRCD1*

**Fig. 1:**
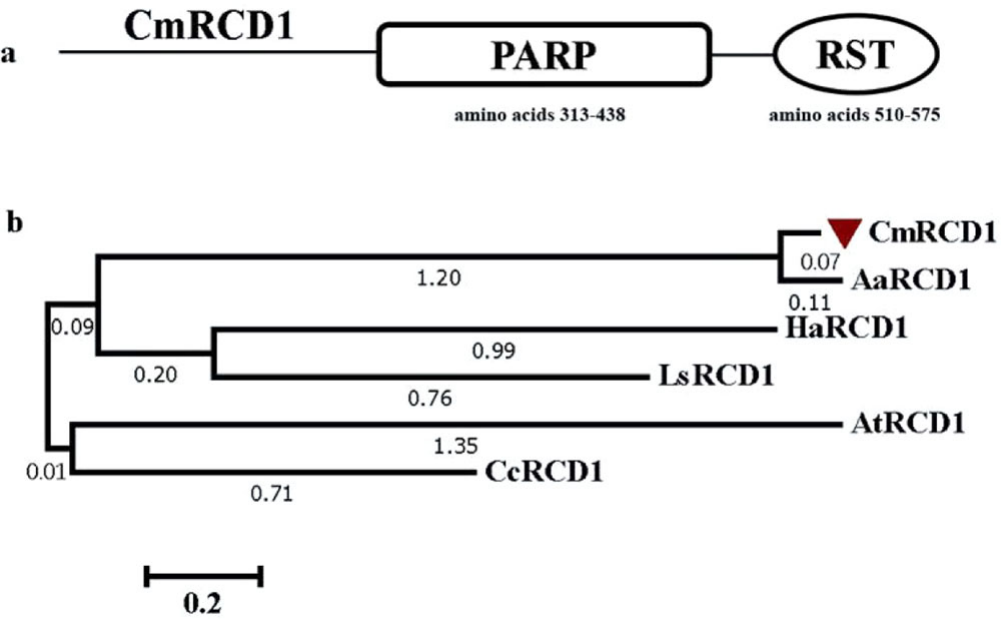
Structure and evolutionary tree of the CmRCD1 protein. **a** Predicted protein structure. **b** Evolutionary tree of RCD1 in chrysanthemum and other plant species. AaRCD1: *Artemisia annua* RCD1 (PWA95938.1); HaRCD1: *Helianthus annuus* RCD1 (XP_021969868.1); LsRCD1: *Lactuca sativa* RCD1 (XP_023736213.1); AtRCD1: *Arabidopsis thaliana* RCD1 (AT1G32230.1); CcRCD1: *Cynara cardunculus* RCD1 (XP_024993358.1)

To investigate the biological functions of CmRCD1 in chrysanthemum, a *CmRCD1* sequence was isolated from ‘Yuuka’ with primers designed in Primer 5.0 that amplified a 1728 bp open-reading frame (ORF) predicted to encode a 575-residue polypeptide consisting of the PARP (amino acids 313–438) and RST (amino acids 510–575) domains (Fig. 1a). Phylogenetic analysis confirmed a close relationship between the ‘Yuuka’ RCD1 and *Artemisia annua L*. RCD1 (Fig. 1b) after amino acid alignment (Fig. S1). Because CmBBX8 has been found to exhibit diurnally controlled expression in our previous research^6^ and because CmRCD1 may interact with CmBBX8, the transcript levels of *CmRCD1* under the regulation of a diurnal clock were further investigated. RNA was extracted from ‘Yuuka’ leaves, and the plants were grown for 2 weeks under long-day (LD) conditions, short-day (SD) conditions, continuous illumination (LL) conditions or continuous darkness (DD) conditions for 48 h. The results showed that the expression of *CmRCD1* was not controlled by photoperiod rhythm (Fig. S2).

### CmRCD1 interacted with CmBBX8

**Fig. 2:**
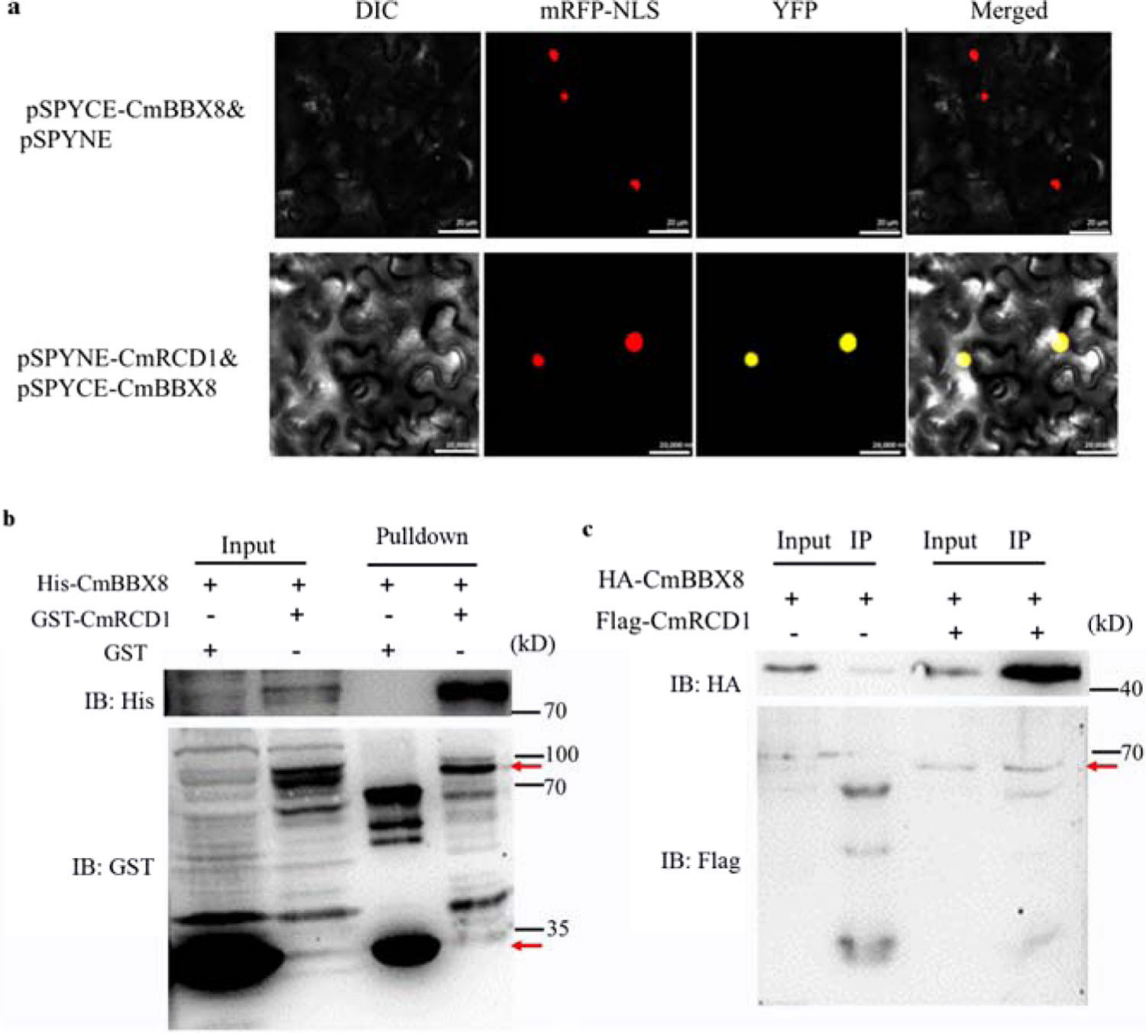
CmRCD1 physically interacted with CmBBX8. **a** BiFC assay. DIC: images taken in bright light; mRFP-NLS: images taken in the red fluorescence channel; YFP: images taken in the yellow fluorescence channel; merged: both overlay plots; bars = 20 μm. **b** Pulldown and **c** CoIP analyses of the interaction between CmRCD1 and CmBBX8. The arrows show the target protein sizes. GST-empty vector & His-CmBBX8 was used as a control for the pulldown assay, and HA-CmBBX8 alone was used as a control for the CoIP assay. The red arrow indicates the size of the target protein

Next, the subcellular localization of CmRCD1 was investigated by transiently expressing the gene in tobacco. Green fluorescent protein (GFP) activity was observed in both the cytoplasm and the nucleus in tobacco cells transiently transformed with the p35S::GFP control plasmid (Fig. S3). The GFP signal overlapped with that of the nuclear marker D53-mCherry in tobacco cells when CmRCD1 was transiently expressed and fused with GFP driven by the CaMV35S promoter (Fig. S3), suggesting that CmRCD1 may localize in the nucleus. A BiFC assay was used to detect the interaction between CmRCD1 and CmBBX8 in tobacco leaves; Yellow fluorescent protein (YFP) activity and the nuclear marker overlapped differently in the experimental plasmids than for the empty vector (EV) control (Fig. 2a). The interaction between CmRCD1 and CmBBX8 was further verified using a pulldown assay. His-CmBBX8, a GST EV and GST-CmRCD1 were all detected as input: the GST-fusion proteins were detected using anti-GST, and the His-fusion proteins were detected using anti-His. The results indicated that these proteins were present in the assay (Fig. 2b). The signal of GST protein with the His-fusion CmBBX8 protein was used as a negative control. Only the His-CmBBX8 protein was immunodetected using anti-His when GST-RCD1 was used as bait in the pulldown assay. The GST empty protein had no His-CmBBX8 protein signal. These results showed that GST-CmRCD1 alone pulled down His-CmBBX8 (Fig. 2b). Moreover, Flag-CmRCD1 was immunoprecipitated by HA-CmBBX8 when transiently coexpressed in tobacco leaf cells (Fig. 2c). Together, these results suggest that CmRCD1 interacts with CmBBX8 both in vivo and in vitro.

### CmRCD1 interacted with the B-box domain of CmBBX8 through its RST domain

**Fig. 3:**
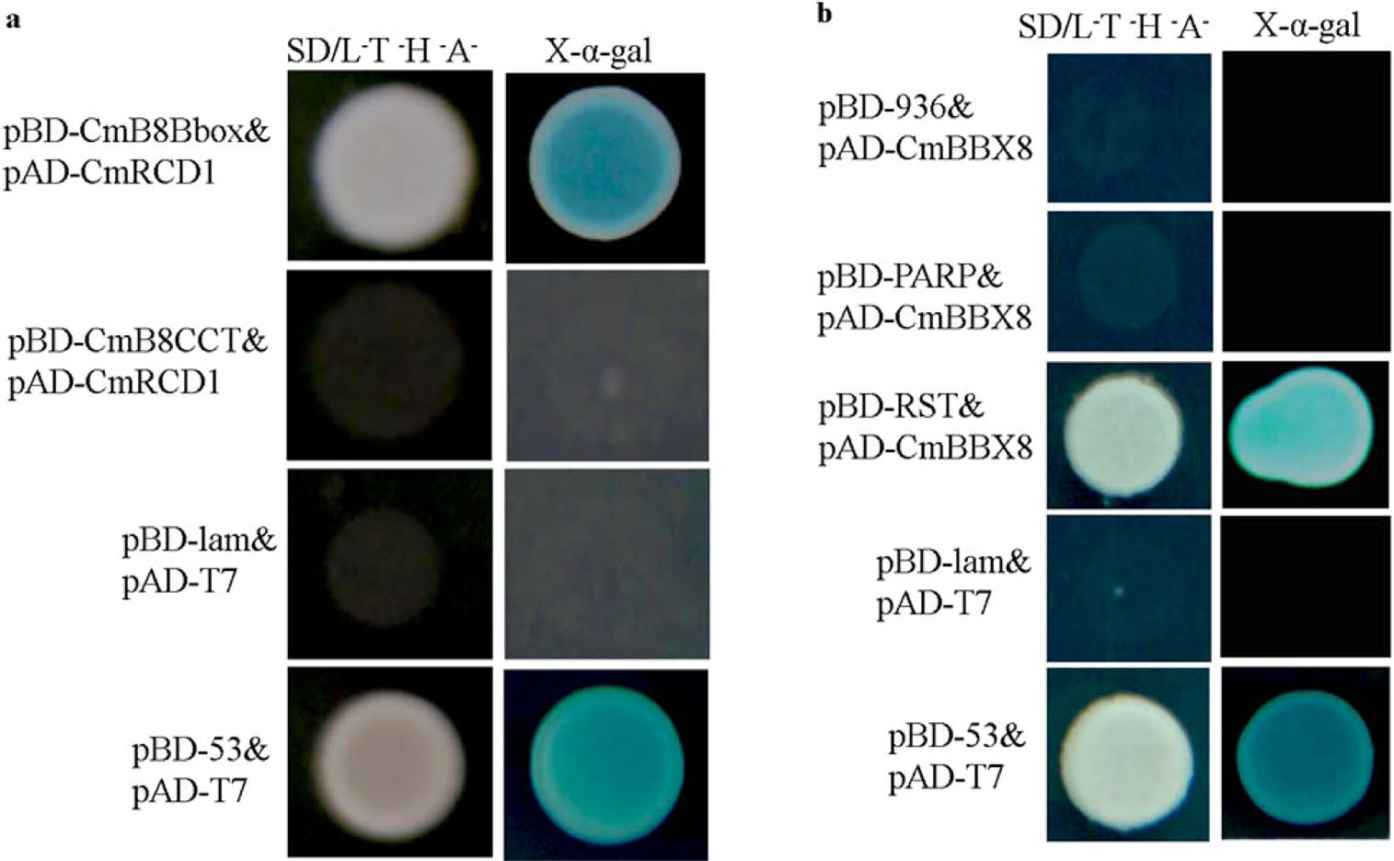
The RST domain of CmRCD1 and the B-box domain of CmBBX8 were required for the CmRCD1-CmBBX8 interaction in yeast cells. p53&pT7 and pT7&plam were used as the positive and negative controls, respectively. **a** Interaction between the segments of CmBBX8 and CmRCD1. **b** Interaction between the segments of CmRCD1 and CmBBX8

Homology modeling analysis using AtRCD1 as a template on the Swiss-Model website revealed that the CmRCD1 protein lacked PARP catalytic activity due to the loss of the Gly-347, Leu-348, and Ser-375 donor sites (Fig. S4). These data indicated that the PARP domain was not involved in the catalysis of CmBBX8. In a yeast two-hybrid assay, when different domains of RCD1 were truncated, the RST domain of CmRCD1 was found to physically interact with the B-box domain of CmBBX8: the yeast grew well on SD/Trp^−^Leu^−^His^−^Ade^−^ medium and exhibited blue coloration on SD/Trp^−^Leu^−^His^−^Ade^−^medium containing X-α-Gal (Fig. 3a). Therefore, the RST domain was shown to be the key structure of the interaction (Figs. S3b and S5).

### CmRCD1 associated with CmBBX8 to repress the upregulation of *CmFTL1*

**Fig. 4:**
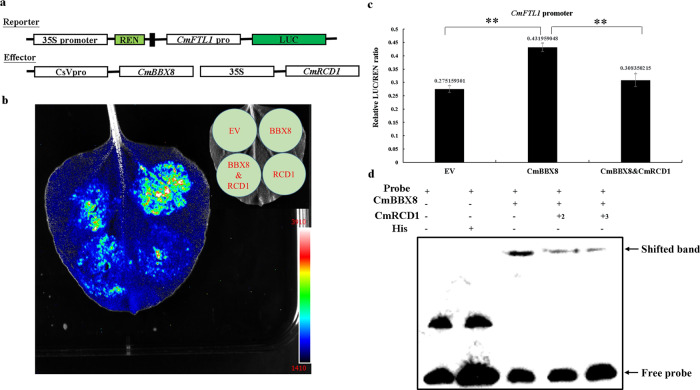
LUC assays and EMSAs verified the mechanism of interaction between CmBBX8 and CmRCD1. **a** Structure of the vector. **b** Antagonistic effects of CmRCD1 on CmBBX8 activation of the *CmFTL1* promoter; from blue to red, the fluorescence value increases gradually. **c** Relative LUC/REN ratios in *Arabidopsis* protoplasts. Student’s *t* test was employed; ** indicates a highly significant difference (*P* < 0.01), and the error bars indicate the SEs (*n* = 3). **d** EMSA results. The two left lanes represent the free combination reactions with the biotin-labeled probe and the His-tag with the biotin-labeled probe, the third lane represents the CmBBX8 protein with the biotin-labeled probe, the fourth lane represents the CmBBX8 protein with the biotin-labeled probe and 2 μL of CmRCD1 protein, and the fifth lane represents the CmBBX8 protein with the biotin-labeled probe and 3 μL of CmRCD1 protein

CmBBX8 acts as a flowering inducer to regulate the expression of *CmFTL1*^6^. To clarify the function of CmRCD1 in the pathway, reporter and effector vectors were constructed (Fig. 4a). A LUC detection assay of tobacco was used to characterize the effects of CmRCD1 and CmBBX8 on the *CmFTL1* promoter based on a previously described method^20^. Compared with the empty vector (EV), the CmBBX8 protein was more capable of activating LUC driven by *CmFTL1* promoter, while the opposite results were observed for the CmRCD1 protein, and the presence of CmRCD1 reduced the activating effect of CmBBX8 (Fig. 4b). To confirm the above results, Renilla luciferase (REN) was used as an internal reference according to a previously described method^20^, and an *Arabidopsis* protoplast transfection experiment showed that CmRCD1 could indeed significantly reduce the ability of CmBBX8 to activate LUC under the control of the *CmFTL1* promoter (Fig. 4c). In addition, an EMSA was used to characterize the DNA-protein interactions involving CmBBX8 and CmRCD1. Consistent with the findings of a previous study^6^, the CCT domain of CmBBX8 was able to bind to the *CmFTL1* promoter subfragment. The binding ability of CmBBX8 protein was decreased in the presence of CmRCD1 protein. As the amount of CmRCD1 increased in the reaction, the CmBBX8-binding affinity decreased (Fig. 4d). Overall, these results indicate that CmRCD1 interferes with CmBBX8 binding to the *CmFTL1* promoter.

**Fig. 5:**
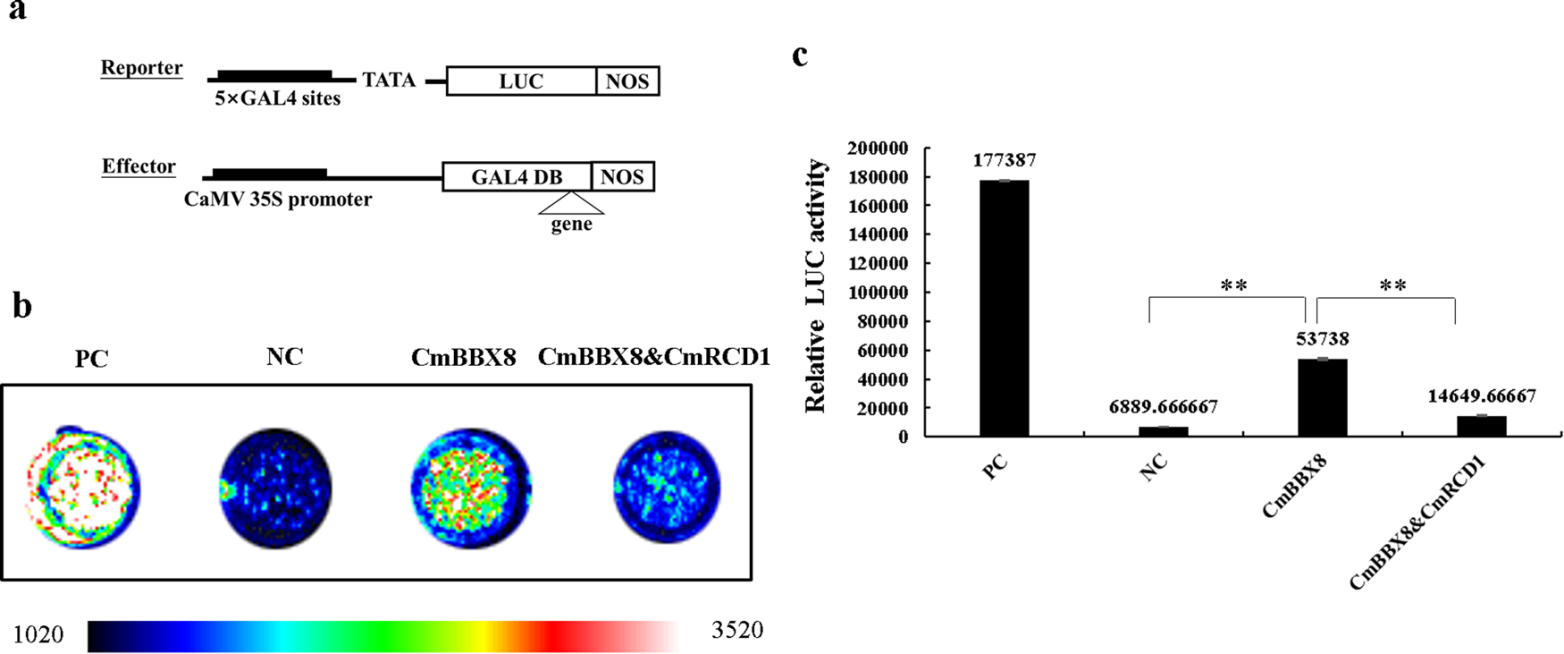
CmRCD1 repressed CmBBX8 activity. PC: positive control (AtARF5); NC: negative control (empty vector). **a** Structure of the vector. **b** Fluorescence images; from blue to red, the fluorescence value increases gradually. **c** Relative LUC activity. The error bars indicate the SEs (*n* = 3); **represents a highly significant difference (Student’s *t* test) (*P* < 0.01)

As the RST domain participates in the assembly of the multimeric general transcription factor complex TFIID^19^, we investigated whether CmRCD1 affected the transcriptional activation function of CmBBX8. An *Arabidopsis* protoplast transfection experiment showed that the transcriptional activation function of CmBBX8 was inhibited by CmRCD1 (Fig. 5a, b). In addition, the relative LUC activity of CmRCD1’s participation was significantly reduced when CmBBX8 was co-expressed (Fig. 5c). Therefore, these data indicate that CmRCD1 also negatively affected the transcriptional activation function of CmBBX8 through its RST domain, thereby repressing the expression of *CmFTL1*.

### CmRCD1 delayed flowering in chrysanthemum

**Fig. 6:**
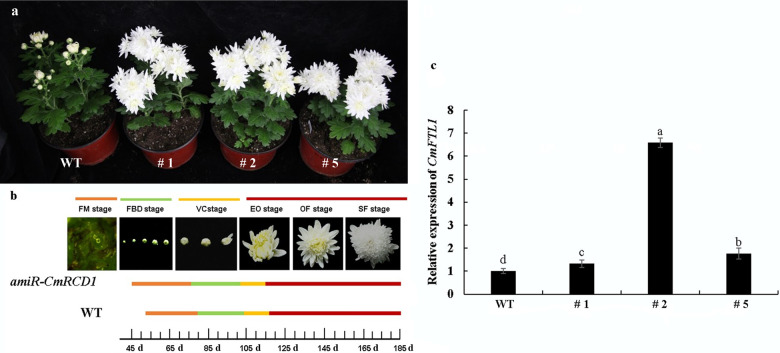
Phenotype of amiR-*CmRCD1* transgenic ‘Yuuka’ at the flowering stage. Wild type: WT; #1, #2, #5: amiRNA plants. **a** Flowering phenotype. **b** Statistics of flowering time. FM: Floral meristem stage; FBD: Flower bud development stage; VC: Visible color stage; EO: Early opening stage; OF: Opened flower stage; SF: Senescing flower stage. **c** Relative expression of *CmFTL1* in amiR-*CmRCD1* plants. Tukey’s honestly significant difference (HSD) test was employed. Different letters above the bars indicate significant differences (*P* < 0.05); the error bars indicate the SEs (*n* = 3)

To determine whether CmRCD1 participates in the genetic regulation of flowering time, *CmRCD1* was specifically knocked down using an artificial *microRNA*^21^. The amiR-*CmRCD1* plants initiated their first involucral primordia 45 days after transplanting, while initiation of the primordia of WT (Wild Type) plants was delayed by approximately one week (Fig. 6a, b). Furthermore, *CmFTL1* was upregulated in amiR-*CmRCD1* transgenic plants (Fig. 6c). These data indicate that *CmRCD1* delayed flowering through downregulation of *CmFTL1*.

## Discussion

CO/BBX1 is a key component of photoperiodic flowering in *Arabidopsis*^22^. In addition to CO/BBX1, multiple other BBX proteins have been shown to play critical roles in the flowering of different plant species. Here, we revealed that CmRCD1 formed inactive heterodimers with CmBBX8, which is a direct activator of *CmFTL1*. The CmRCD1-CmBBX8 regulatory module modulated *CmFTL1* transcription by fine-tuning the initiation of flowering in chrysanthemum.

*Arabidopsis* RCD1 possesses a WWE domain, a PARP-like (poly [ADP-ribose] polymerase-like) domain, and an RST domain at the C-terminal region^14^. RCD1 plays pleiotropic roles in various developmental processes and in response to diverse abiotic and biotic stresses in plants^13,21,23^. A yeast two-hybrid assay revealed that RCD1 interacts with a large number of transcription factors, such as ethylene responsive factors (AP2/ERF), NAC family transcription factors (NAM, ATAF1/2 and CUC2) and basic Helix-Loop-Helix (bHLH) transcription factors^13^. A growing number of studies have shown that RCD1 interacts with the transcription factors ANAC013^24^, DREB2A^25^ and Rap2.4a^26^, and affects their transcriptional activation function. These facts suggest that RCD1 functions as a transcriptional regulator by directly associating with multiple transcription factors and affecting their behavior. Via yeast assays, an interaction between RCD1 and COL9 (BBX7) has been found, but the mechanism is unknown^13^. CmBBX8 directly binds to the promoters of *CmFTL1* to activate its expression, thereby promoting flowering^6^. Here, we found that CmRCD1 negatively controlled flowering via a similar molecular mechanism: CmRCD1 physically interacted with CmBBX8 to affect its biochemical activity, consequently inhibiting the expression of *CmFTL1* and flowering in chrysanthemum (Figs. 4 and 5).

BBX protein-mediated floral initiation is a central theme of light-dependent plant development. CO-FT represents a key regulatory hub of flowering control, and this module is evolutionally conserved in various plant species^27,28^. CO associates with the CORE cis-element present in the *FT* promoter region to upregulate its expression, enabling the accumulation of FT. Accumulated foreign FT in the leaves shifts to the shoot apex, where it initiates floral development^29^. CO, which is a B-box-containing protein, directly controls FT at the transcriptional level. To maintain a proper FT level, a set of factors converge on CO to modulate its activity. For instance, BBX10, BBX19 and TOEs mediate CO activity through direct protein-protein interactions^1,2,30^; this appears to be a common regulatory mode for control of CO activity.

The function of the CmBBX8-CmFTL1 module is critical for initiation of flowering in chrysanthemum. Our previous studies have shown that CmBBX8 directly associates with the CORE cis-element (CCACA) within the *CmFTL1* promoter and activates its transcription. CmFTL1, a close ortholog of FT, accelerates flowering in chrysanthemum grown under LD conditions. CmRCD1 negatively controls the initiation of flowering through the CmBBX8-CmFTL1-mediated pathway and represses the binding of CmBBX8 to the *CmFTL1* promoter by directly interacting with CmBBX8. These molecular events consequently lead to inhibition of *CmFTL1* at the mRNA level and to repression of flowering in chrysanthemum. Thus, the CmRCD1-CmBBX8-CmFTL1 signaling pathway may precisely control flowering in chrysanthemum in response to dynamically changing environmental conditions, which is potentially relevant for chrysanthemum molecular breeding strategies.

## Materials and methods

### Isolation of CmRCD1 and analysis of its structure

The *CmRCD1* ORF sequence was amplified using the primer pair CmRCD1-F/-R (annexed table, Wang et al., 2020^21^). The conserved domains were analyzed using the NCBI database (www.ncbi.nlm.nih.gov/Structure/cdd/wrpsb.cgi). The phylogeny of a set of RCD1 sequences recovered from GenBank was derived using MEGA7.0 software with the neighbor-joining algorithm and 1000 bp replications^31^.

### Yeast two-hybrid experiments

To introduce the pENTR1A-CmBBX8 construct, the primer set CmBBX8 (*Bam*H I)-F/CmBBX8 (*Eco*R I)-R (annexed table) was used and recombined into the final pGADT7 vector using the LR recombination reaction. The CmBBX8-Bbox and CCT segment sequences were amplified using primer pairs designed to incorporate an *Eco*R I site at one end of the amplicon and a *Bam*H I site at the other end (annexed table). *Bam*H I/*Eco*R I digestion and ligation of the pGBKT7 and amplified segments were then performed. The CmRCD1-PARP, RST and 936-segment sequences were amplified by using primer pairs designed with the CE Design app (annexed table) and by subsequently conducting a separate homologous recombination reaction with pGADT7. The resulting constructs were transferred into the yeast strain *Y2H*. Yeast cells were cultured on synthetic dropout (SD)/Leu^−^Trp^−^medium for 3 days at 30°C and then transferred to SD/His^−^Ade^−^Leu^−^Trp^−^plates in either the presence or absence of X-α-Gal.

### Bimolecular fluorescence complementation assay and luciferase assay

The *CmRCD1* and *CmBBX8* sequences were amplified using the primer pairs CmBBX8 YFP-F/CmBBX8 YFP-R and CmRCD1 YFP-F/CmRCD1 YFP-R (annexed table) with *Bam*H I and *Kpn* I and were inserted into the pSPYNE/YCE vector, which contains a reporter gene encoding YFP^32^. The *A. tumefaciens* strain *GV3101* harboring CmBBX8 and CmRCD1 was grown to an OD_600_ of 0.5, and infiltration medium (10 mM MES, 200 μM AS, 10 mM MgCl_2_) was introduced via syringe into the leaves of a 5-6-week-old *Nicotiana benthamiana* plant. After 48–96 h, a confocal laser scanning microscope (ZEISS, LSM780) was used to observe the YFP signal according to a previously described method^33^, and a CCD camera (Tanon 5200) was used to observe luciferase activity following a published method^34^.

### Pulldown assay

To introduce the pENTR1A-CmRCD1 construct, the primer set CmBBX8 (*BamH* I)-F/CmBBX8 (*EcoR* I)-R (annexed table) was used and recombined into the final vector pDEST-15 (GST tag) using the LR recombination reaction. pDEST-15-*CmRCD1*, together with His-CmBBX8 (*Kpn* I/*Pst* I) (annexed table) constructed previously, was transfected into *BL21 E. coli* to induce protein expression. Next, 20 μL of prewashed GST magnetic beads (Promega, Wisconsin-Madison) were added to the protein-containing supernatant and incubated at 4°C overnight. WB detection was performed with a His antibody (Thermo, USA)^35^.

### Coimmunoprecipitation assay

pCsVMV-HA3-N-1300-CmBBX8 was constructed in a homologous recombination reaction, and pENTR1A-CmRCD1 was recombined into the final vector pEarleyGate202-CmRCD1 (Flag tag) using the LR recombination reaction. The *A. tumefaciens* strain *GV3101* harboring the constructs pEarleyGate202-CmRCD1 and pCsVMV-HA3-N-1300-CmBBX8 was introduced into *N. benthamiana* plants. After 48–96 h, the leaves were sampled, and proteins were extracted with EB buffer (100 mL: 0.4 M sucrose, 1 mL of 1 M Tris-HCl pH 8.0, 35 µL of 14.3 M stock β-Me, 100 µL of 100 mM PMSF, 2 cocktail tablets). The protein was first incubated with 20 μL of HA beads at 4°C overnight (Thermo, USA) and then incubated with a Flag antibody for WB detection^36^.

### Transient activation experiments in protoplasts

GALDB4-CmRCD1 and GALDB4-CmBBX8 were constructed according to the above LR recombination method. Protoplasts were prepared from 3-week-old *Columbia* WT Arabidopsis plants by affixing the epidermal cells with scotch tape, immersing them in an enzymolysis solution (20 mL: 1 mL of 100 mM MES, 1.44 g of d-mannitol, 0.2 g of cellulase R10, and 0.02 g of pectinase R10) at 55°C for 10 min, and then cooling the solution to 25°C. Next, 1.6 mL of 100 mM CaCl_2_ and 0.03 g of BSA were added at 28°C, and enzymatic hydrolysis was conducted for 1.5–2 h at 50 rpm until the tape became transparent. Then, the 35-mesh nylon membrane was rinsed with W5 buffer (100 mL: 0.9 g of NaCl, 1.84 g of CaCl_2_, 5 mL of 100 mM KCl, and 2 mL of 100 mM MES), and the protoplasts were filtered under a microscope. The protoplasts were then slowly washed with W5, and 1 mL of MMg buffer (10 mL: 0.729 g of mannitol, 1.5 mL of 100 mM MgCl_2_, 400 μL of 100 mM MES) was gently added. After immersion in an ice bath for 30 min, 10 µg of plasmids were transfected into 100 μL of protoplasts with 110 μL of PEG and W5 to stop the reaction^37^. The solution was incubated under light for 16 h, and 7.8 mM sodium fluorescein was added. The relative LUC and REN activity was measured with a GloMax (GloMax^®^ 20/20), and photographs were taken with a CCD camera (Tanon 5200).

### EMSA

Protein expression and biotin probe application were conducted according to the protocols of the pulldown assay and Wang et al. (2020)^6^, respectively. The subsequent EMSAs were performed using a Light Shift^TM^ Chemiluminescent EMSA Kit (Thermo Fisher, New York) following the manufacturer’s protocol^6^.

### qRT-PCR analysis

RNA extraction, reverse transcription, and qRT-PCR were conducted following the manufacturer’s protocol^6^. The primer pairs (qCmRCD1-F/qCmRCD1-R, qCmFTL1-F/qCmFTL1-R) used to amplify CmBBX8 and CmRCD1 cDNA are given in the annexed table. Estimates of transcript abundance were calculated using the method published by Livak and Schmittgen^38^. The reference sequence was chrysanthemum *EF1a* (KF305681.1). Tukey’s honestly significant difference test and Student’s *t* test were employed; a difference was considered significant at *P* < 0.05 or *P* < 0.01 for all data. For rhythm expression analysis, before the plants had formed 15–19 fully expanded leaves, they were held for 2 weeks under LD conditions. The plants were then transplanted into a cabinet supplying a 16 or 8 h photoperiod with continuous illumination and DD conditions. RNA was extracted from the third leaf from the shoot tip of cv. ‘Yuuka’.

### Quantification of flowering time

A microscope was used to recognize when chrysanthemum had entered the flowering transition stage and EB stage, at which point at least 50% of the ray flowers had unfolded^39^. Statistics were tracked for the following periods: the FBD stage, VC stage, EO stage, OF stage, and SF stage. Measurements were taken from at least 20 plants^7^.

### 3D homology modeling

The catalytic activity of CmRCD1 was analyzed in the Swiss-Model database (https://www.swissmodel.expasy.org/) with AtRCD1 as the homology template.

## Supplementary information

Supplemental
