# Supplementary material for: CmRCD1 represses flowering by directly interacting with CmBBX8 in summer chrysanthemum
Source: Hortic Res. 2021 Apr 1;8:79. doi: 10.1038/s41438-021-00516-z (PMC8012346; doi:10.1038/s41438-021-00516-z)
Supplement: Supplementary file 1 — Supplemental [file 41438_2021_516_MOESM1_ESM.docx]

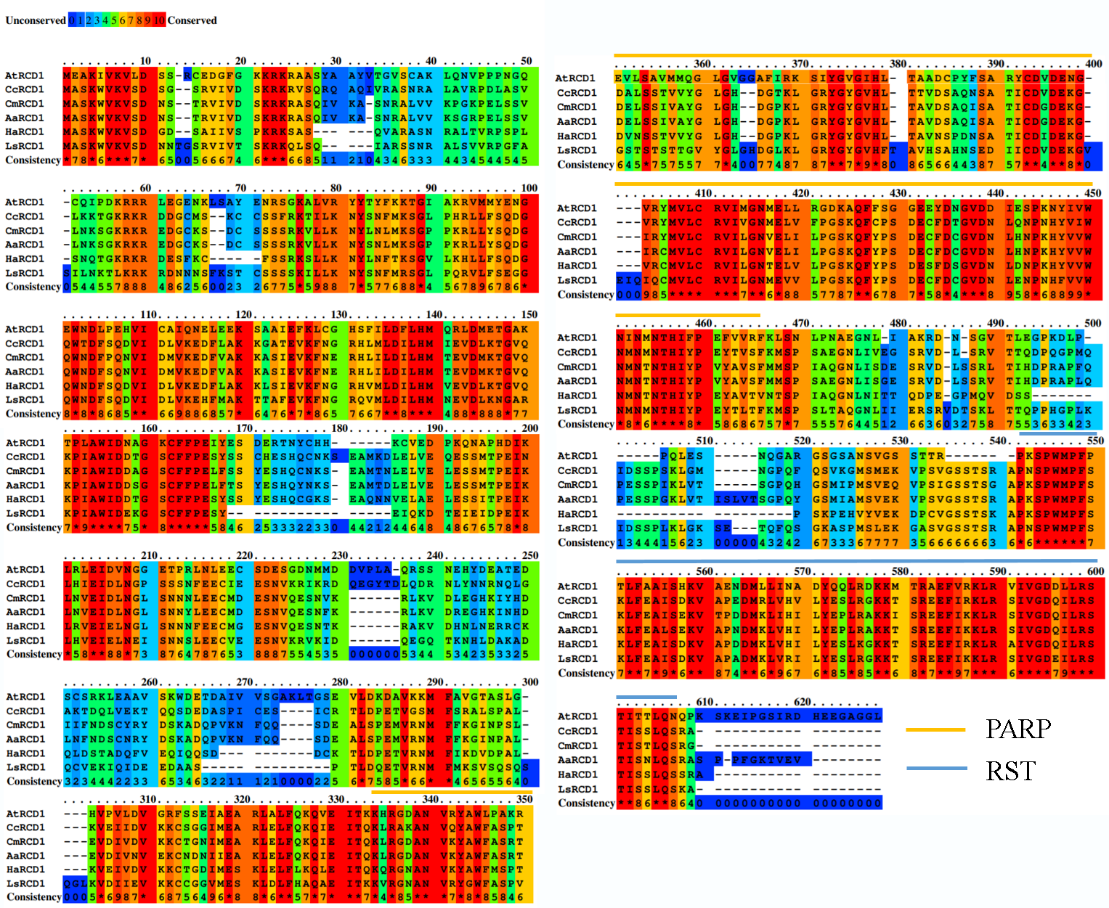


Figure S1. Homologous comparison of CmRCD1 protein with the homologous protein from other plant species. AtRCD1 (Arabidopsis thaliana RCD1, AT1G32230.1), CcRCD1 (Cynara cardunculus RCD1, XP_024993358.1), AaRCD1 (Artemisia annua RCD1, PWA95938.1), HaRCD1 (Helianthus annuus RCD1, XP_021969868.1), LsRCD1 (Lactuca sativa RCD1, XP_023736213.1).


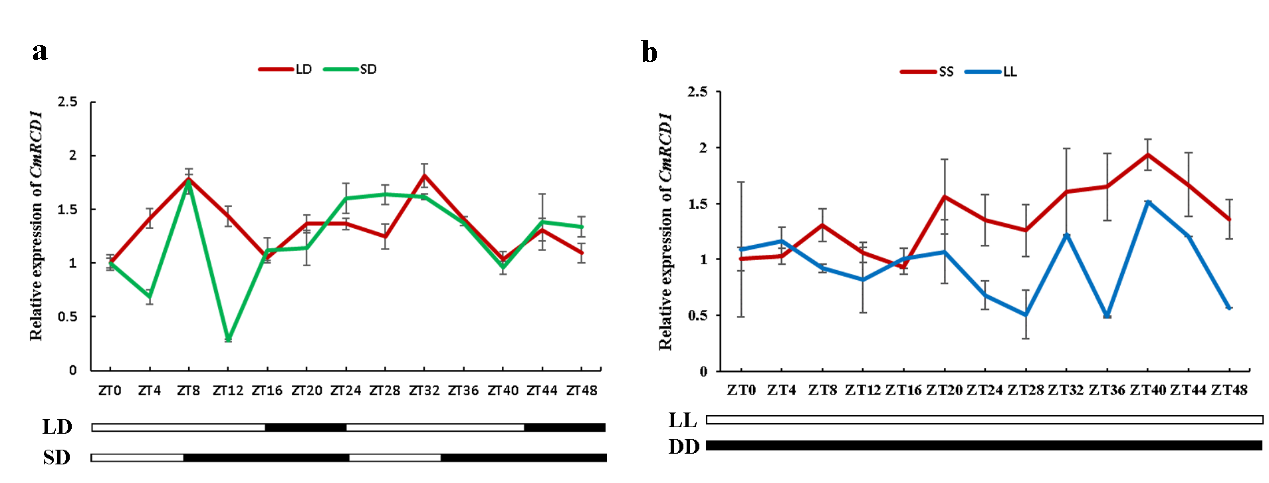


Figure S2. The transcriptional response to photoperiod. (a) LD (long-day) and SD (short-day) conditions; (b) LL (continuous illumination) and DD (continuous darkness) conditions. The abscissa indicates the sampling time point; white and black horizontal bars below the axis represent light and dark periods, respectively. The error bars represent the SE.


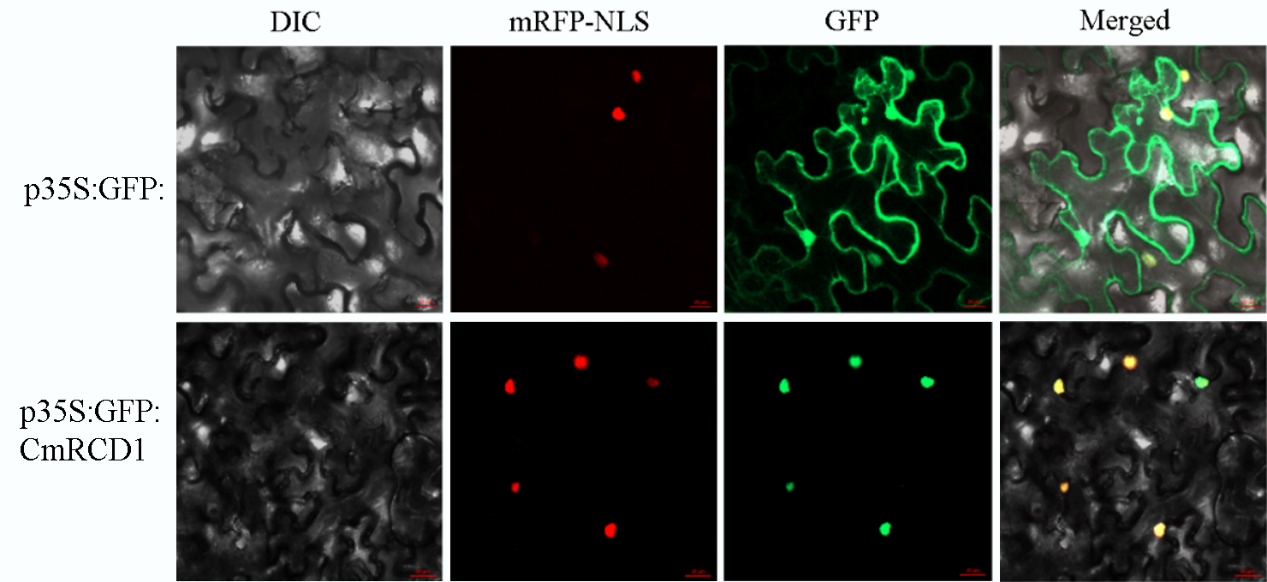


Figure S3. Subcellular localization of CmRCD1 in *N. benthamiana*. mRFP-NLS: photos in the red fluorescence channel; GFP: photos in the green fluorescence channel; DIC: photos in bright light; merged: both overlay plots; bars = 30 μm.


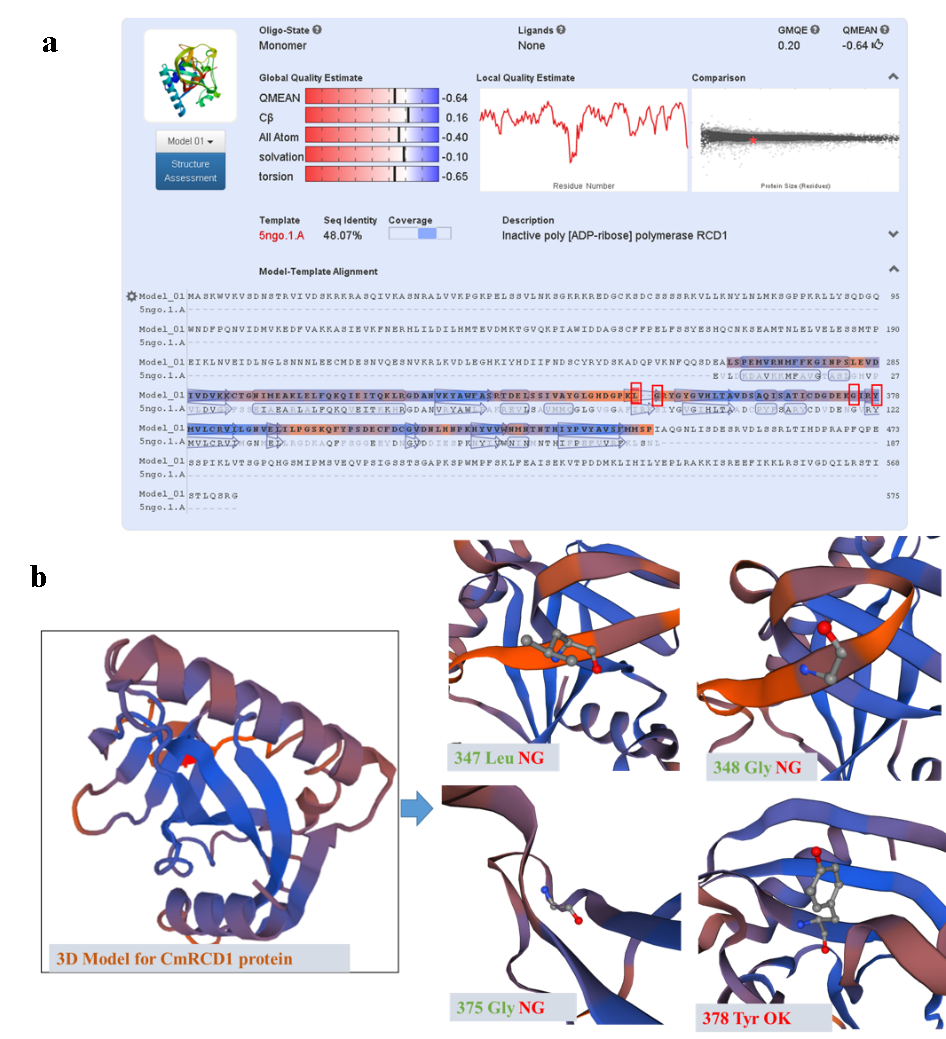


Figure S4. Catalytic activity analysis of the CmRCD1 protein. Red box: parameter reliability analysis and protein secondary structure; green box: three-level structure of CmRCD1.


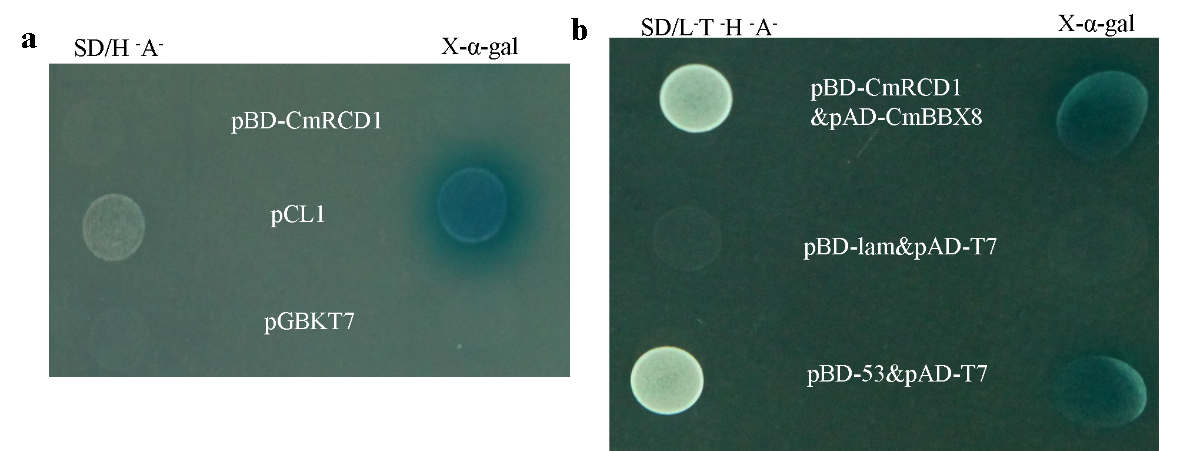


Figure S5. The interaction between CmRCD1 and full-length CmBBX8 in yeast cells. (a) Transactivation activity analysis of CmRCD1 in yeast cells. pCL1 and an empty pGBKT7 were used as the positive and negative controls, respectively; (b) analysis of the interaction of CmRCD1 and CmBBX8 in yeast cells. p53&pT7 and pT7&plam were used as the positive and negative controls, respectively.
